# Overview of the SARS-CoV-2 genotypes circulating in Latin America during 2021

**DOI:** 10.3389/fpubh.2023.1095202

**Published:** 2023-03-02

**Authors:** Jose Arturo Molina-Mora, Jhonnatan Reales-González, Erwin Camacho, Francisco Duarte-Martínez, Pablo Tsukayama, Claudio Soto-Garita, Hebleen Brenes, Estela Cordero-Laurent, Andrea Ribeiro dos Santos, Cláudio Guedes Salgado, Caio Santos Silva, Jorge Santana de Souza, Gisele Nunes, Tatianne Negri, Amanda Vidal, Renato Oliveira, Guilherme Oliveira, José Esteban Muñoz-Medina, Angel Gustavo Salas-Lais, Guadalupe Mireles-Rivera, Ezequiel Sosa, Adrián Turjanski, María Cecilia Monzani, Mauricio G. Carobene, Federico Remes Lenicov, Gustavo Schottlender, Darío A. Fernández Do Porto, Jan Frederik Kreuze, Luisa Sacristán, Marcela Guevara-Suarez, Marco Cristancho, Rebeca Campos-Sánchez, Alfredo Herrera-Estrella

**Affiliations:** ^1^Centro de investigación en Enfermedades Tropicales and Facultad de Microbiología, Universidad de Costa Rica, San José, Costa Rica; ^2^Grupo de Genómica de Microorganismos Emergentes, Instituto Nacional de Salud, Bogotá, Colombia; ^3^Investigaciones Biomédicas, Universidad de Sucre, Sincelejo, Colombia; ^4^Laboratorio de Genómica y Biología Molecular, Instituto Costarricense de Investigación y Enseñanza en Nutrición y Salud, Tres Ríos, Cartago, Costa Rica; ^5^Facultad de Ciencias y Filosofía, Universidad Peruana Cayetano Heredia, Lima, Peru; ^6^Instituto de Ciências Biológica, Universidade Federal do Pará, Belém, Brazil; ^7^Instituto Metrópole Digital, Universidade Federal do Rio Grande do Norte, Natal, Brazil; ^8^Environmental Genomics, Vale Institute of Technology, Belém, Pará, Brazil; ^9^Coordinación de Calidad de Insumos y Laboratorios Especializados, Instituto Mexicano del Seguro Social, Ciudad de Mexico, Mexico; ^10^Laboratorio Nacional de Genómica para la Biodiversidad-Unidad de Genómica Avanzada, Centro de Investigación y de Estudios Avanzados, Irapuato, Mexico; ^11^Facultad de Ciencias Exactas y Naturales, Universidad de Buenos Aires, Buenos Aires, Argentina; ^12^Consejo Nacional de Investigaciones Científicas y Técnicas (CONICET), Buenos Aires, Argentina; ^13^Facultad de Medicina de la Universidad de Buenos Aires, Buenos Aires, Argentina; ^14^International Potato Center, Lima, Peru; ^15^Vicerrectoria de Investigación y Creación, Universidad de Los Andes, Bogotá, Colombia; ^16^Centro de Investigación en Biología Celular y Molecular, Universidad de Costa Rica, San José, Costa Rica

**Keywords:** COVID-19, Latin America, SARS-CoV-2, genomic surveillance, CABANA, coronavirus

## Abstract

Latin America is one of the regions in which the COVID-19 pandemic has a stronger impact, with more than 72 million reported infections and 1.6 million deaths until June 2022. Since this region is ecologically diverse and is affected by enormous social inequalities, efforts to identify genomic patterns of the circulating SARS-CoV-2 genotypes are necessary for the suitable management of the pandemic. To contribute to the genomic surveillance of the SARS-CoV-2 in Latin America, we extended the number of SARS-CoV-2 genomes available from the region by sequencing and analyzing the viral genome from COVID-19 patients from seven countries (Argentina, Brazil, Costa Rica, Colombia, Mexico, Bolivia, and Peru). Subsequently, we analyzed the genomes circulating mainly during 2021 including records from GISAID database from Latin America. A total of 1,534 genome sequences were generated from seven countries, demonstrating the laboratory and bioinformatics capabilities for genomic surveillance of pathogens that have been developed locally. For Latin America, patterns regarding several variants associated with multiple re-introductions, a relatively low percentage of sequenced samples, as well as an increment in the mutation frequency since the beginning of the pandemic, are in line with worldwide data. Besides, some variants of concern (VOC) and variants of interest (VOI) such as Gamma, Mu and Lambda, and at least 83 other lineages have predominated locally with a country-specific enrichments. This work has contributed to the understanding of the dynamics of the pandemic in Latin America as part of the local and international efforts to achieve timely genomic surveillance of SARS-CoV-2.

## Introduction

In December 2019, several cases of a new respiratory illness were described in Wuhan, China. About a month later, it was confirmed that the illness COVID-19 (coronavirus disease 2019) was caused by a novel coronavirus which was subsequently named SARS-CoV-2 (1, 2). Until June 2022, the COVID-19 pandemic had impacted the world with >549 million confirmed cases of COVID-19, including >6.3 million deaths. Latin America was one of the most strongly impacted regions with more than 72 million reported infections and >1.6 million deaths during the same period.

SARS-CoV-2 genome sequences have been reported from many regions of the world and these data have been proven useful in tracking the global spread of the virus. Genomic epidemiology of SARS-CoV-2 has shed light on the origins of regional outbreaks, global dispersal, and epidemiological history of the virus (3, 4). Until April 2022, over 11.5 million genomes had been deposited in the GISAID database (https://www.gisaid.org/), out of which >376,000 were reported by Latin American countries.

Since its appearance, a large genetic diversity has been recognized for SARS-CoV-2 due to widespread transmission and geographical isolation (5). The emergence of new genotypes (lineages, clades, variants, etc.) is the product of a natural process that occurs when viruses replicate at high rates as it happens during a pandemic (4). The World Health Organization (WHO) has classified five divergent genotypes as variants of concern (VOC: Alpha, Beta, Gamma, Delta, Omicron), as well as some lineages into variants of interest (VOI: Lambda, Mu, Epsilon, Zeta, Theta, Iota, Eta, Kappa, and others) and variants under monitoring (VUM: B.1.640 and XD) (6). All reported variants and other lineages have been identified in Latin America (7), including genotypes that were first reported regionally, such as Gamma in Brazil, Mu in Colombia, and Lambda in Peru (6), as well as unique lineages in Costa Rica and Central America (8, 9). Those descriptions of locally enriched genotypes exemplify the opportunities that SARS-CoV-2 has found in Latin America for spreading and evolving. This scenario is in part explained by the complex environmental and human reality in this region, with huge ecological diversity and social inequalities (10, 11). Thus, efforts on revealing the behavior of SARS-CoV-2 are necessary to identify regionally emerging patterns for the suitable management of the pandemic, which cannot be inferred from North America, Europe, or Asia (11).

In this context, the CABANA initiative (Capacity building for Bioinformatics in Latin America, Global Challenges Research Fund GCRF: www.cabana.online) supported the development of a regional project titled “The SARS-CoV-2 genome, its evolution and epidemiology in Latin America” during 2021. The project had the direct participation of seven institutions from Argentina, Brazil, Bolivia, Colombia, Costa Rica, Mexico, and Peru. Efforts of this project included not only the sequencing and genome assembly of the SARS-CoV-2 virus from a total of 1,534 COVID-19 cases in those countries, but also to bring a more complete overview of the SARS-CoV-2 genotypes circulating in Latin America during 2021 using public databases. Thus, this study aimed to contribute to the genomic surveillance of the SARS-CoV-2 to understand the dynamics of the pandemic in Latin America by providing genome sequences and analyzing circulating genotypes during the year 2021.

## Methods

### Samples and ethical considerations

Respiratory samples were obtained from public and private laboratories belonging to the national network of SARS-CoV-2 diagnostics in each country. Adequate transportation and storage conditions were guaranteed to preserve the samples. Every sample was anonymized to protect patients' identity. Being a notifiable disease, the metadata was collected from the forms that accompanied the samples, either in the national reference laboratories or in the ministries of health. See Supplementary material for IDs to access metadata in the GISAID database.

### Sample sequencing and genome analysis

To contribute with SARS-CoV-2 genome sequences from Latin America, seven participant countries (Argentina, Bolivia, Brazil, Costa Rica, Colombia, Mexico, and Peru) were involved in sample processing from COVID-19 patients. Diagnosis using RT-qPCR, genome sequencing and assembly, as well as genotyping, were implemented using the laboratory protocols and bioinformatic pipelines that are being locally used as part of the genomic surveillance efforts in each country as shown in Table 1 and reported in (9, 12, 13). Genome sequences were uploaded to the GISAID database (https://www.gisaid.org/). Details regarding the number of processed samples (assembled genomes), laboratory and bioinformatic protocols for each country are summarized in Table 1. GISAID accession numbers (ID) for assembled genomes are presented in Supplementary material.

**Table 1:** Sequencing strategy and bioinformatic pipelines used for the genomic surveillance of the SARS-CoV-2 in five Latin American countries, CABANA initiative.

**Country**	**Number of samples**	**Participant institutions**	**Sequencing protocol**	**Bioinformatic pipeline**
Argentina	220	Universidad de Buenos Aires (UBA) and Consejo Nacional de Investigaciones Científicas y Técnicas (CONICET)	Illumina platform: Nextera XT DNA library and MiSeq sequencer (Illumina, San Diego, CA, USA).	Genome assembly, variant calling, and genotyping: Custom protocol with BWA-MEM/Freebayes/SNPEff.
Bolivia	94	Laboratorio de diagnóstico e investigación BIOSCIENCE SRL and Laboratorio de Genómica Microbiana, Universidad Peruana Cayetano Heredia	Illumina platform: Illumina COVIDSeq Test and Illumina NextSeq 550 sequencer (Illumina, Inc., San Diego, CA, USA).	Genome assembly, variant calling, and genotyping: Illumina DRAGEN COVID Lineage v3.5.3 BaseSpace App.
Brazil	167	Vale Institute of Technology, Belém, PA, Brazil	Illumina platform, following a custom protocol and Illumina NextSeq sequencer.	Genome assembly, variant calling, and genotyping: PipeCoV pipeline described in (12).
Costa Rica	190	Universidad de Costa Rica (UCR) and Instituto Costarricense de Investigación y Enseñanza en Nutrición y Salud (INCIENSA)	Illumina platform, following the protocol described in (9, 13) with a Illumina DNA Prep Kit/Nextera DNA flex library and MiSeq sequencer (Illumina, San Diego, CA, USA).	Genome assembly, variant calling, genotyping, and phylogeny: Described in (9)
Colombia	147	Universidad de Los Andes.	Nanopore platform with a GridION sequencer.	Genome assembly: custom protocol for long-reads sequencing with Minimap2/Nanopolish. Variant calling, genotyping, and phylogeny: (9)
Mexico	472	Unidad de Genómica Avanzada del Centro de Investigación y de Estudios Avanzados (UGA-LANGEBIO, CINVESTAV)	Illumina platform: Illumina COVIDSeq test and Illumina MiSeq sequencer (Illumina, Inc., San Diego, CA, USA).	Genome assembly, variant calling, genotyping, and phylogeny: Described in (9)
Peru	244	Laboratorio de Genómica Microbiana, Universidad Peruana Cayetano Heredia	Illumina platform: Illumina COVIDSeq Test and Illumina NextSeq 550 sequencer (Illumina, Inc., San Diego, CA, USA).	Genome assembly, variant calling, and genotyping: Illumina DRAGEN COVID Lineage v3.5.3 BaseSpace App.

### Analysis of circulating SARS-CoV-2 genotypes in Latin America

To gain insights into the SARS-CoV-2 genotypes circulating in Latin America during 2021, a general analysis was done using the genome sequences available at the GISAID database (https://www.gisaid.org/). Selection of countries, statistics of sequenced samples, and plots of circulating genomes and mutation frequency were obtained using the tools of the GISAID platform. The number of COVID-19 cases per country was retrieved from the daily reports of the Pan American Health Organization (14). All analyses were performed considering sequences collected until January 31th, 2022. PANGOLIN lineage database (15, 16) was used to analyze the frequency of lineages among countries.

## Results and discussion

Genomic surveillance has been a hallmark of the COVID-19 pandemic that, in contrast to other pandemics, achieves tracking of the virus evolution and spread worldwide almost in real-time (4).

In this work, we extended the repertoire of SARS-CoV-2 genome sequences with a total of 1,534 sequences from seven Latin American countries (Table 1). Whereas, this was a relatively modest contribution to the overall quantity of sequences produced in this period in Latin America for certain time-intervals and countries it provided important complementarity for the genomic surveillance of the virus. In Bolivia for example, our efforts represented 38% of all sequences produced over this time. To perform a more complete examination, we included all sequences from Latin America available at the GISAID database collected up to January 2022. A total of 221,228 genomes sequences, including the 1,534 provided by this work, were analyzed by genotype and the mutation profile.

According to the GISAID database records, the numbers of sequences is still small in comparison to the number of diagnosed cases in Latin America (Table 2). On average, only 0.39% of COVID-19 cases in Latin America had been sequenced, with Mexico and Chile having the highest rates with 0.98 and 0.92%, respectively. In the case of Nicaragua, in which the pandemic has been downplayed (17, 18), the reports of diagnosed patients and other statistics are considered unrealistic, including the 2.92% of sequenced samples. Thus, we did not conduct comparisons of Nicaragua among other countries due to the extremely biased data.

**Table 2:** Comparison of COVID-19 cases and sequenced samples among Latin American countries from the beginning of the pandemic to January 31th, 2022.

**Country**	**Population**	**Total COVID-19 cases**			**Sequenced samples**	
		**Absolute number**	**Percentage of the population (%)**	**Deaths**	**Absolute number**	**Percentage of COVID-19 cases (%)**
Mexico	131,026,542	4,942,590	3.77	306,091	48,329	0.98
Chile	19,369,864	2,190,561	11.31	39,733	20,086	0.92
Belize	408,778	50,487	12.35	625	376	0.74
Ecuador	18,057,002	732,038	4.05	34,533	3,770	0.52
Peru	33,681,601	3,239,538	9.62	205,834	14,790	0.46
Brazil	214,895,351	25,454,105	11.84	627,589	101,532	0.40
Costa Rica	5,166,024	694,865	13.45	7,575	2,719	0.39
El Salvador	6,536,807	135,109	2.07	3,899	308	0.23
Colombia	51,721,161	5,887,261	11.38	13,400	12,520	0.21
Panama	4,419,710	700,274	15.84	7,732	1,223	0.17
Guatemala	18,427,485	690,290	3.75	16,385	1,420	0.21
Paraguay	7,267,941	583,662	8.03	17,321	882	0.15
Uruguay	3,492,345	668,425	19.14	6,479	717	0.11
Argentina	45,836,859	8,378,656	18.28	121,273	11,553	0.14
Honduras	10,147,994	391,874	3.86	10,504	116	0.03
Venezuela	28,311,334	485,974	1.72	5,447	123	0.03
Bolivia	11,919,079	855,705	7.18	20,951	248	0.03
Nicaragua	6,746,365	17,650	0.26	216	516	2.92
Latin America	617,432,242	56,099,064	9.09	1,445,587	221,228	0.39
Worldwide	7,900,000,000	380,099,991	4.81	5,695,345	7,748,697	2.04

On the other extreme, Bolivia, Honduras and Venezuela have barely sequenced even 0.03% of samples derived from all patients diagnosed with the disease. There is no single Latin American country that has sequenced more samples, relative to the number of cases reported, than the world average that corresponds to 2.04%, which is low too. The current scenario is congruent with a previous report with < 0.5% of sequenced samples for Latin American countries (19). These findings represent not only part of the regional disparities in the SARS-CoV-2 genomic surveillance efforts in Latin America, but also that this geographic region needs to increase the effort to achieve the sequencing of at least 5% of positive samples to detect emerging viral lineages when their prevalence is < 1% of all strains in a population, as suggested previously (20). In fact, globally, only 6.8% of 189 countries around the world reached this value (19). This situation is like that of other latitudes around the world in which only a very small portion of the countries has reached the recommended percentage, suggesting that sequencing at least 0.5% of the cases, with a time in days between sample collection and genome submission < 21 days, could be a benchmark for SARS-CoV-2 genomic surveillance efforts for low- and middle-income countries (19) taking into account the high cost of sequencing reagents and equipment in these countries. In high income countries, around 25% of the genomes were submitted within 21 days, contrasting with the pattern observed in 5% of the genomes from low- and middle-income countries. Thus, the identification of patterns regarding the circulating genotypes in Latin America should be interpreted with cautions due the differences of SARS-CoV-2 surveillance systems, including sequencing capacity and sampling strategies between countries in the region.

**Figure 1:**
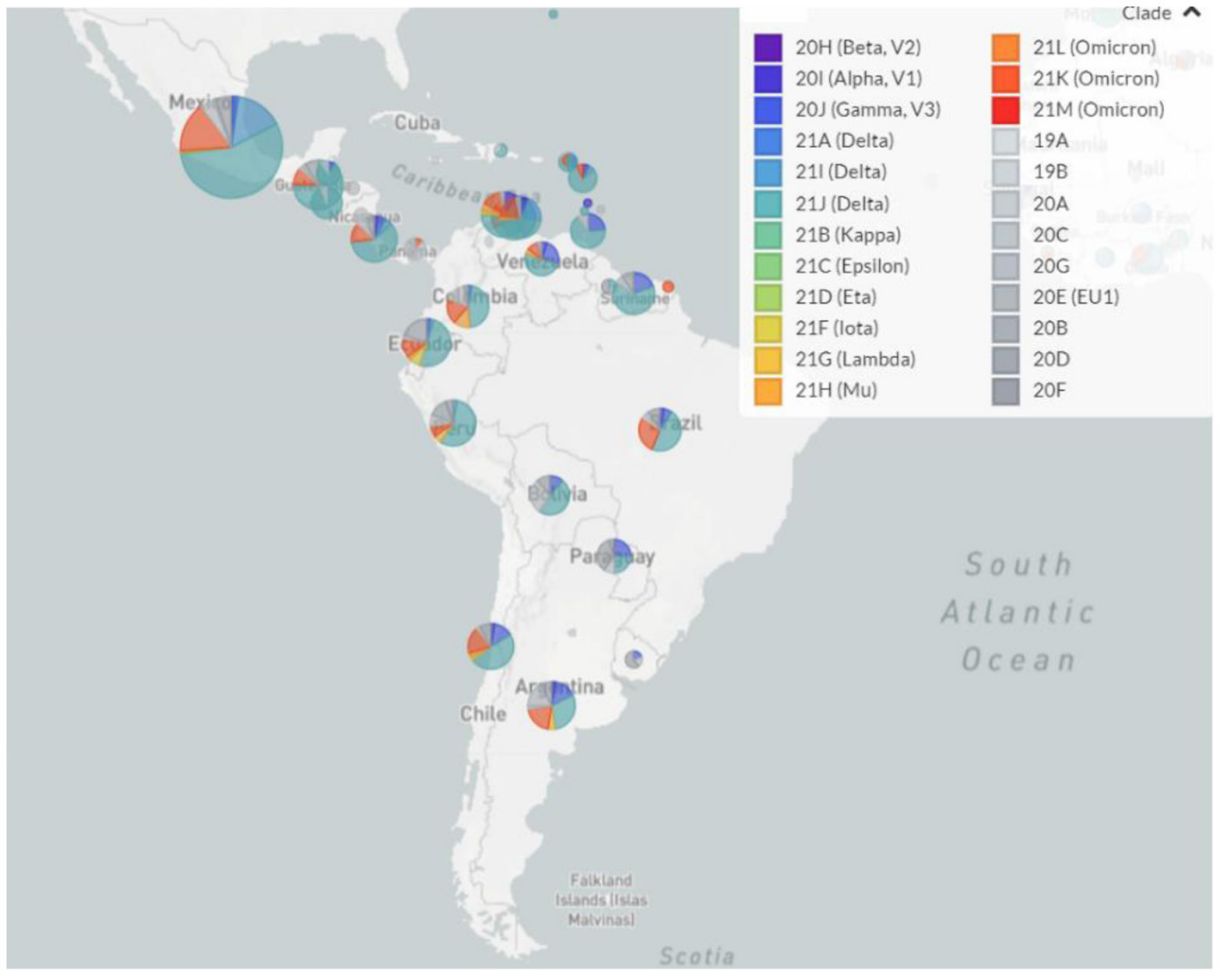
Landscape of the SARS-CoV-2 genotypes circulating in Latin America from February 2021 to January 2022. Pie charts indicate the relative abundance of distinct SARS-CoV-2 genotypes in each country.

**Figure 2:**
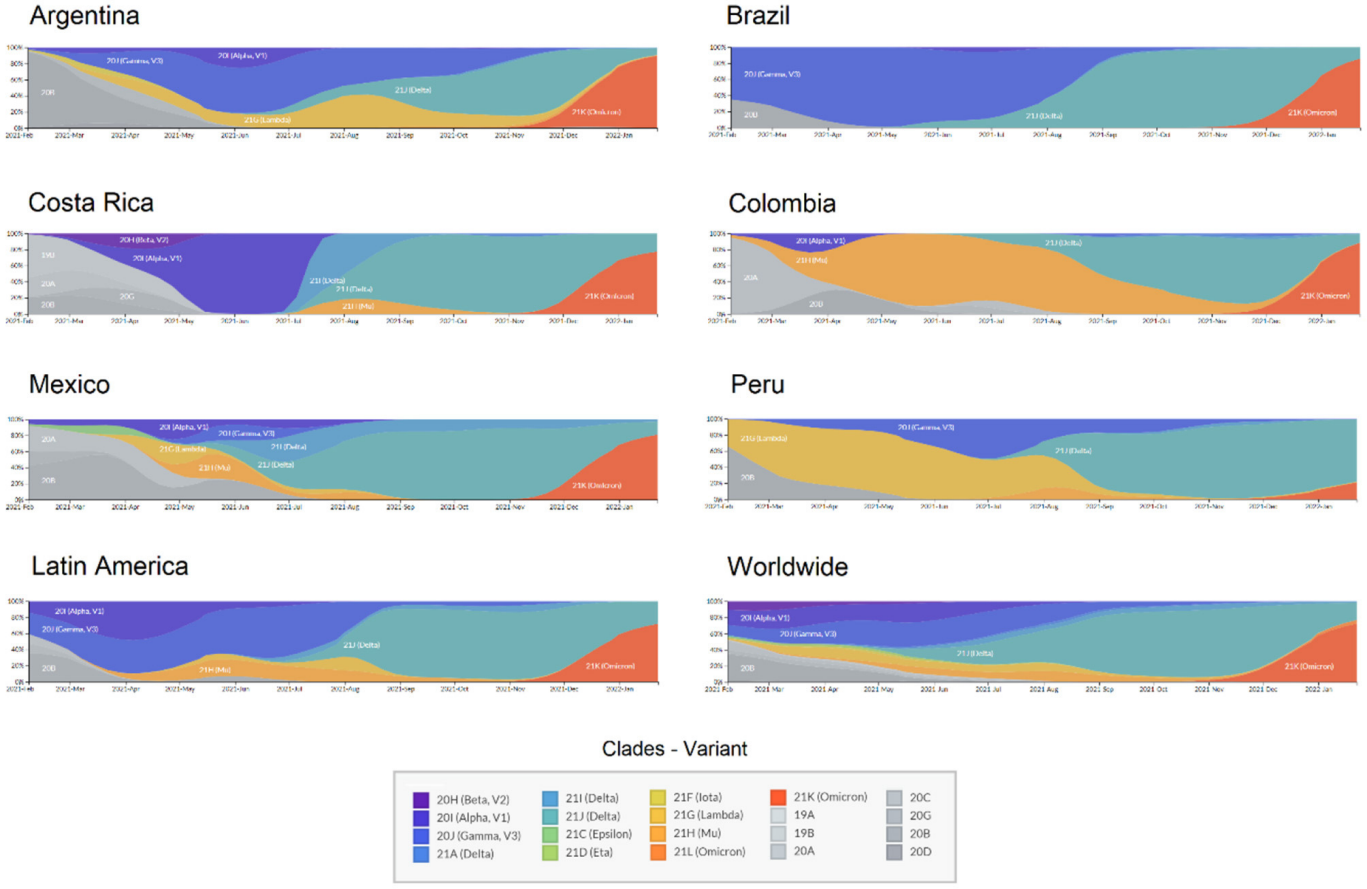
Transition of SARS-CoV-2 variants circulating in six Latin American countries and worldwide during 2021. X: time from February 2021 to January 2022; Y: Relative abundance of SARS-CoV-2 genotypes.

Regarding the circulating genotypes, the reports on the diversity of lineages are similar to other studies in Latin America (9, 21–23) and other distant geographic regions (24, 25). For divergent SARS-CoV-2 genomes, all VOCs have been reported in all Latin American countries, resulting in a large diversity of genotypes circulating in each country (Figure 1). This is in line with the expected pattern of multiple and independent re-introductions due to population mobility within Latin America, as well as to and from other countries and continents (26–28). Besides, some genotypes have been reported with an epicenter in Latin America. As presented in Figure 2, those country-specific variants were predominant in the first semester of 2021, such as the Gamma variant in Brazil until August 2021, the Mu variant in Colombia from April to September 2021, and Lambda in Peru during the period from March to June 2021 (29). Other remarkable genome versions were the case of the Gamma variant predominating between June and August 2021 in Argentina, as well as the more mixed pattern with distinct variants in Mexico, similar to the average for the entire Latin American region. In comparison to the rest of the world, Latin America reported similar transitions between the Alpha, Delta, and Omicron variants. Nonetheless, the increased reports of Mu and Lambda in this region were minimal for the worldwide representation (Figure 2).

In Latin America recurrent dissemination of SARS-CoV-2 through shared borders between countries has been evidenced (30), allowing rapid entrance and dissemination of different lineages to the different countries (31). Territories with no restriction to international interchange are more likely to introduce multiple SARS-CoV-2 variants, including variants of concern and/or interest and even lineages with mutations of concern and emerging variants with different mutation patterns (32). These introductions of VOCs to Latin America were more evident during the second half of the year 2021, where the Delta variant displaced other variants in several countries and became predominant as shown in Figure 2, while during the first semester of the year lineage predominance varied among these countries.

Although several epidemiological aspects can be associated with these patterns, the extensive opening of the borders during the middle of 2021 possibly favored the spread of new variants of concern in the region. Besides, the presence of multiple mutations that have been associated with increased infectivity and/or escape from immune response in variants such as Delta (33) helped this variant to displace other variants, as it occurred worldwide.

For other genotypes, at least 83 out of >1,500 PANGOLIN lineages have been reported with a high predominance in a Latin American country (Table 3). The full list of lineages is presented in the Supplementary material. As an example, lineage C.39 was predominant in Chile with 45.0% of all the sequences reported, followed by France (15.0%), Peru (12.0%), Guinea (10.0%), and Germany (8.0%). From these lineages, at least 80% of the sequences from 51 lineages have been reported to come from a Latin American country (Tables 3, 4). In the distribution by country, Brazil, Peru, Chile, Costa Rica, and Mexico have more reports of lineages with a frequency >80% locally.

**Table 3:** Number of lineages in which a Latin American country is predominant by frequency.

**Country**	Lineages in which the country is predominant^*^	Lineages in which the country has frequency >80%^**^
Argentina	4	3
Brazil	30	21
Chile	11	6
Colombia	2	0
Costa Rica	6	3
Ecuador	1	0
Mexico	5	2
Panama	1	1
Peru	21	13
Uruguay	2	2
Total	83	51

* Refers to those lineages in which the main source of sequences is from a Latin American country. See text for details.

** Similar to the previous case, but lineages are only counted if the predominant country has a percentage >80%. See text for details.

**Table 4:** Lineages reported with a frequency >80% among Latin American countries.

**Lineage**	**Most common countries**	**First detected—country**	**Total sequences worldwide**
A.2.4	Panama 96.0%, Costa_Rica 2.0%, Northern_Mariana_Islands 1.0%, United States of America 1.0%, United Kingdom 0.0%	Panama	291
C.4	Peru 95.0%, United States of America 2.0%, Turkey 2.0%, Republic of Serbia 1.0%, Switzerland 1.0%	Peru	130
C.11	Chile 97.0%, Peru 2.0%, Argentina 1.0%	Chile	95
C.13	Peru 84.0%, United States of America 14.0%, Japan 2.0%	Peru	45
C.14	Peru 92.0%, United States of America 3.0%, Japan 2.0%, Democratic Republic of the Congo 1.0%, Brazil 1.0%	Peru	253
C.25	Peru 100.0%	Peru	8
C.29	Chile 89.0%, Russia 6.0%, Denmark 6.0%	Chile	18
C.32	Peru 95.0%, Turkey 2.0%, Denmark 2.0%, United Kingdom 2.0%	Peru	57
C.33	Peru 90.0%, Japan 5.0%, United States of America 5.0%	Peru	20
C.40	Peru 97.0%, Chile 3.0%	Peru	37
P.1.3	Brazil 100.0%	Brazil	29
P.1.4	Brazil 98.0%, Costa_Rica 1.0%, Peru 1.0%, United States of America 0.0%, Colombia 0.0%	Brazil	1039
P.1.5	Brazil 100.0%	Brazil	14
P.1.6	Brazil 99.0%, Sweden 0.0%, French_Guiana 0.0%	Brazil	562
P.1.7	Brazil 91.0%, United States of America 5.0%, Spain 2.0%, Peru 0.0%, Mexico 0.0%	Japan	3666
P.1.7.1	Peru 94.0%, United States of America 2.0%, Brazil 1.0%, Chile 1.0%, Puerto_Rico 0.0%	Bolivia	773
P.1.8	Brazil 94.0%, Sweden 1.0%, France 1.0%, United Kingdom 1.0%, Spain 1.0%	Brazil	257
P.1.9	Brazil 97.0%, United States of America 1.0%, Mexico 1.0%, Belgium 1.0%	Brazil	236
P.1.10.2	Mexico 100.0%	Mexico	23
P.1.11	Brazil 96.0%, Ecuador 2.0%, Spain 1.0%, United States of America 1.0%	Brazil	102
P.1.12.1	Peru 91.0%, United States of America 4.0%, Italy 2.0%, Chile 1.0%, Switzerland 1.0%	Italy	161
P.4	Brazil 100.0%, United States of America 0.0%	Brazil	234
P.5	Brazil 95.0%, Philippines 3.0%, United States of America 2.0%	Brazil	44
P.6	Uruguay 95.0%, Philippines 2.0%, United States of America 1.0%, Norway 0.0%, Spain 0.0%	Uruguay	298
B.1.1.33	Brazil 83.0%, United States of America 5.0%, Chile 3.0%, Argentina 2.0%, Paraguay 1.0%	United States	2130
N.3	Argentina 97.0%, Bolivia 2.0%, Hong_Kong 1.0%, Chile 1.0%	Argentina	124
N.4	Chile 92.0%, Brazil 4.0%, United States of America 1.0%, Peru 1.0%, New_Zealand 0.0%	Canada	232
N.5	Argentina 87.0%, United States of America 7.0%, Spain 1.0%, Italy 1.0%, United Kingdom 1.0%	India	365
N.6	Chile 97.0%, Brazil 1.0%, Japan 1.0%, Paraguay 1.0%	Chile	138
N.7	Uruguay 100.0%	Uruguay	33
N.9	Brazil 96.0%, Ireland 1.0%, Argentina 1.0%, Japan 1.0%, Chile 1.0%	Brazil	138
N.10	Brazil 97.0%, Germany 3.0%	Brazil	22
B.1.1.110	Peru 92.0%, Finland 8.0%	Finland	11
B.1.1.324	Chile 100.0%	Chile	17
B.1.1.332	Brazil 100.0%	Brazil	29
B.1.1.389	Costa_Rica 86.0%, United States of America 6.0%, Spain 5.0%, Finland 2.0%, Australia 1.0%	Costa Rica	139
B.1.1.442	Argentina 80.0%, Turkey 7.0%, United States of America 5.0%, Germany 5.0%, Austria 2.0%	Argentina	47
B.1.1.516	Costa_Rica 100.0%	Nicaragua	21
B.1.110.1	Chile 82.0%, United States of America 18.0%	Chile	12
B.1.205	Peru 98.0%, Israel 2.0%	Peru	40
B.1.243.2	Mexico 81.0%, United States of America 19.0%	Mexico	60
B.1.291	Costa_Rica 94.0%, United States of America 4.0%, Australia 2.0%	Nicaragua	67
AY.25.1.1	Peru 82.0%, United States of America 14.0%, Chile 2.0%, Colombia 1.0%, Switzerland 1.0%	USA	172
AY.26.1	Peru 81.0%, United States of America 12.0%, Mexico 2.0%, Israel 1.0%, Chile 1.0%	Peru	154
AY.43.1	Brazil 96.0%, United Kingdom 1.0%, United States of America 1.0%, France 1.0%, Chile 1.0%	Poland	1036
AY.43.2	Brazil 99.0%, France 0.0%, Japan 0.0%, Belgium 0.0%, Switzerland 0.0%	India	1295
AY.43.7	Brazil 98.0%, Netherlands 1.0%, France 1.0%, Israel 1.0%	France	184
AY.46.3	Brazil 95.0%, United States of America 1.0%, Turkey 1.0%, India 0.0%, Czech_Republic 0.0%	India	1565
AY.99.1	Brazil 85.0%, United Kingdom 10.0%, United States of America 4.0%, India 1.0%, Spain 0.0%	India	1372
AY.99.2	Brazil 97.0%, United States of America 1.0%, Chile 0.0%, France 0.0%, Portugal 0.0%	Brazil	22815
AY.101	Brazil 81.0%, Chile 10.0%, Colombia 4.0%, Peru 2.0%, United States of America 1.0%	Colombia	4300

For instance, Peru had 97.0% of the sequences reported for lineage C.40 and 95% of lineage C.4. Also, Peru was the main country in which the AY.25.1.1 and AY.26.1 genotypes (Delta sub-lineages) were documented. Brazil reported 97% of the 22,815 cases of lineage AY.99.2 (firstly reported in Colombia), that was demonstrated to successfully disseminate among different locations in the country (34, 35). Lineages derived from the Gamma variant were also reported frequently in Brazil (e.g., P.1.4, P.1.7, P.4, and others). During 2020 the lineage B.1.1.389, which harbors the specific mutation spike:T1117, was reported as predominant in Costa Rica (86% of cases of this lineage were reported in this country) (9). Despite its dominance, few changes were predicted on the virus behavior (transmission, immune response, and other) and it was quickly replaced by the lineage Central America and subsequently by VOCs such as Alpha and Gamma (8). In the case of Mexico, two lineages (P.1.10.2 and B.1.243.2) were mainly found in this country (frequency >80%) but in a limited number. Lineage B.1.1.519 was a relevant genotype reported in Mexico, despite it was mainly reported in the United States. This version predominated in Mexico during the first quarter of 2021 while the Alpha variant (B.1.1.7) was also spreading. Interestingly, unlike other cases, the Alpha variant did not displace B.1.1.519 in this country (36). B.1.1.519 was assigned as a VUM by WHO in 02-Jun-2021 and was degraded to a FMV (formerly monitoring variant) on 9-Nov-2021 (https://www.who.int/activities/tracking-SARS-CoV-2-variants).

Jointly, these results indicate that specific mutations and the subsequent consolidation into lineages were detected in Latin America and evidenced by genomic surveillance in the region. Interestingly, 17 of these lineages were first reported in a different country from where it was subsequently found to be predominant (>80%). This includes neighboring countries, such as the case of lineage P.1.7.1 which was enriched in Peru but was first reported in Bolivia. This pattern was more frequent for Brazil, with eight lineages that were first reported in other countries including from Europe and Asia, but that became dominant in this country.

Tracking of specific mutations into Latin American lineages that could be used as local markers, may help to identify transmission networks locally and globally, highlighting the need for each country and territory to strengthen the sequencing and bioinformatic capacities. These capacities can also be of use to locally study other scenarios such as clinical profiles for COVID-19 patients (37), immune escape (38), long-term COVID-19 (39), identification of co-infections (40) or identify recombinant genomes (a recognized mechanism of viral diversity in coronaviruses).

**Figure 3:**
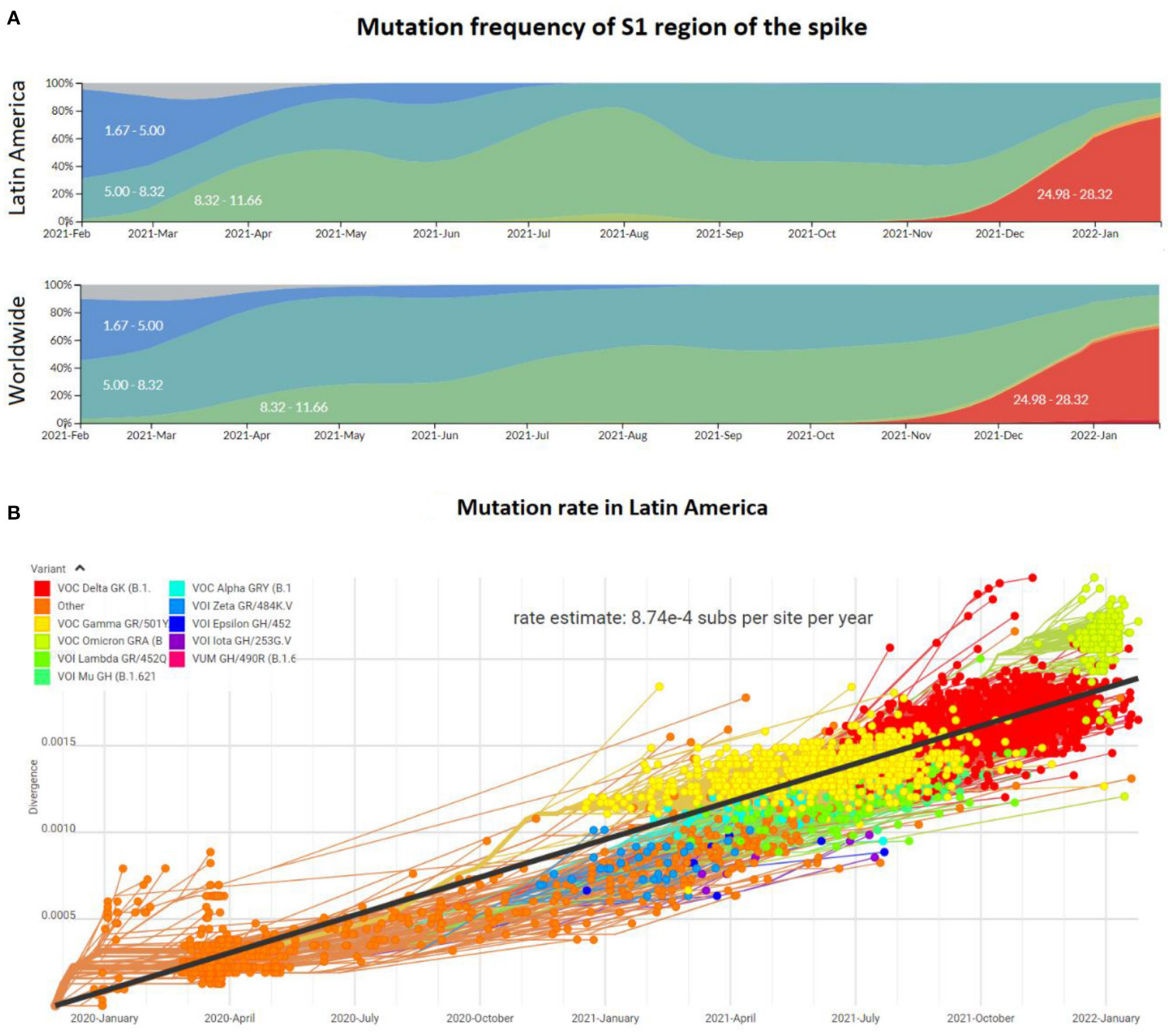
Mutation profile of the SARS-CoV-2 genome in Latin America and worldwide from the beginning of the pandemic to January 2022. **(A)** Mutation frequency in the region S1 encoding the spike protein of the SARS-CoV-2 viral genome. Colors represent the intervals for the absolute mutation number. **(B)** Mutation rate of the SARS-CoV-2 genomes during the pandemic, including distinct genotypes (colors) and the approach to estimate consensus rate (black line).

Despite the reports of differences in the enriched genotypes in the first half of 2021, the emergence of new variants of the viral genome in Latin America was consistent with the rest of the world inferred from the mutation frequency (Figure 3A). During 2020, the mutation frequency for the S1 region of the spike gene was estimated at around 2-3 mutations per month. At the beginning of 2021, this frequency increased to 8.32 and subsequently to around 12 with the predominance of Delta. However, with the arrival of the Omicron variant, the frequency at the very end of 2021 and the first month of 2022 reached values of 28 mutations, in both, Latin America and the world. Thus, this accumulative divergence has impacted the mutation rate over the pandemic, which until January 2022 was estimated to be around 8.74 × 10^−4^ substitutions per site per year (Figure 3B). This mutation frequency and rate values are consistent with other local and global studies during the pandemic (41–43), including the rate of 0.8 – 2.38 × 10^−3^ substitutions per site per year described by Banerjee et al. (44). Following the gradual reopening of borders and worldwide travels, the frequency of infections and the appearance of mutations and new genotypes are expected to increase (45). Thus, more genome sequencing studies, including robust metadata collection, and more financial support are needed to continue with the surveillance of the pandemic in Latin America.

Finally, since most countries in this region are considered low- and middle-income countries, the impact of the COVID-19 pandemic on society has been devastating socially and economically (46). Genomic surveillance is pivotal as a powerful tool for decision-makers regarding the management of the pandemic in the Latin American context concerning social and economic measures, as well as practical decisions in terms of the diagnostic tools, treatments, and vaccines (4). On the other hand, local and prompt reports of emerging genotypes demonstrated the laboratory and bioinformatic capabilities in Latin American countries. These capabilities were developed locally in the last years for the surveillance of pathogens and other applications. Jointly, the local and international efforts to achieve the genomic surveillance of SARS-CoV-2 have contributed to the understanding of the dynamics of the pandemic in Latin America, which is an ongoing process.

In addition, the infrastructure related to molecular diagnostic techniques experimented a relevant advance due to the pandemic. Before the pandemic outbreak, these techniques were only available in advanced clinical laboratories but now an expanded availability and a cost-effective implementation are found in most clinical laboratories toward-becoming routine tests to study other pathogens and diseases (47).

Regarding limitations, the main drawback of this study is that we assumed that all sequences were comparable, with no segregation by experimental or bioinformatic conditions. The GISAID platform accepts a variety of conditions to upload genome sequences without restriction associated with the sample processing strategy, sequencing technology, genome assembler, variant callers and others, which were not considered here to assess their impact on the genotyping. Although previous reports have found differences in the used pipelines (48), we made the analysis using the whole set of available sequences as performed in other studies (19, 49, 50). Also, as an infectious disease, the clinical outcome of COVID-19 depends on the epidemiological triad: (i) environmental conditions (social behavior, restriction measurements, management of cases, others), (ii) host factors (ethnicity, risk factors, genetic profile of HLA or ACE-II alleles, others), and (iii) the virus (genotype and mutations that impact transmission, immune response, others). Here we have only considered the SARS-CoV-2 genotypes in the period but other data associated with the epidemiological triad and change of the circulating versions of the virus is relevant to include in further analyses.

## Conclusions

In conclusion, with this study we have contributed to the genomic surveillance of the SARS-CoV-2 in Latin America by providing 1,534 genome sequences from seven countries and the subsequent global analysis of circulating genomes mainly during 2021. For Latin America, patterns regarding several variants associated with multiple re-introductions, a relatively low proportion of sequenced samples, as well as an increase in the mutation frequency, are in line with worldwide data. Additionally, some genotypes such as Gamma, Mu and Lambda variants and 83 lineages have emerged locally with a subsequent country-specific predominance. Regional efforts demonstrate the laboratory and bioinformatics capabilities for the genomic surveillance of pathogens that have been developed in Latin America, and which is expected to continue during the current COVID-19 pandemic.

## Data availability statement

The datasets presented in this study can be found in online repositories. The names of the repository/repositories and accession number(s) can be found in the article/Supplementary material.

## Ethics statement

Ethical review and approval was not required for the study on human participants in accordance with the local legislation and institutional requirements. Written informed consent for participation was not required for this study in accordance with the national legislation and the institutional requirements.

## Author contributions

JM-Mo, PT, GO, JK, MC, RC-S, and AH-E participated in the conception and design of this study. FD-M, CS-G, HB, EC-L, JM-Me, AV, AR, CG, CS, JS, AS-L, LS, MG-S, and GM-R were involved in sample processing. JM-Mo standardized the bioinformatics pipelines and drafted the manuscript. JM-Mo, GN, RO, TN, JR-G, and EC were involved in data analysis. JM-Mo, FD-M, GO, JK, MC, RC-S, and AH-E participated in the interpretation of results. All authors reviewed and approved the final manuscript.
